# Reproducible testing for embedded BCIs: a demultiplexing PCB and acquisition system for EEG signal emulation

**DOI:** 10.1016/j.ohx.2026.e00800

**Published:** 2026-05-27

**Authors:** Daniel Enériz, Diego Antolín, Nicolás Medrano, Belén Calvo

**Affiliations:** Group of Power Electronics and Microelectronics (GEPM), Aragon Institute for Engineering Research (I3A), University of Zaragoza, Zaragoza, Spain

**Keywords:** BCI, EEG, Emulation, ADC

## Abstract

Validating machine learning models for Brain-Computer Interfaces (BCIs) on resource-constrained edge devices is challenging, as traditional methods rely on costly EEG equipment or simulations that fail to capture real-world electronic characteristics. To bridge this gap, we introduce the DEEGMUX, a low-cost, open-source hardware system for high-fidelity, hardware-in-the-loop (HIL) testing of EEG classification algorithms. The system comprises an EEG Demultiplexer Board that converts a multiplexed EEG signal into 8 parallel channels, and an EEG Acquisition and Processing Board featuring an ADS1299 24-bit ADC interfaced with an Arduino Nano 33 BLE. This setup enables the use of real EEG datasets, such as the PhysioNet Motor Imagery dataset, to generate precisely timed electronic signals. Characterization demonstrated high signal fidelity, with a Mean Squared Error of 1.7·10^−10^ V^2^ and a Signal-to-Noise Ratio of 16 dB relative to the original digital data. Furthermore, an EEGNet motor imagery classifier evaluated on hardware-acquired signals showed a negligible accuracy difference of (−0.3 ± 5)% compared to evaluation on the original data, confirming that the emulation chain preserves classification-relevant features. The DEEGMUX provides a scalable, reproducible, and affordable platform for rigorously testing edge-deployed CNN models against realistic electronic inputs, accelerating the transition from simulation to robust real-world BCI deployment.

## Graphical abstract

**Figure:**
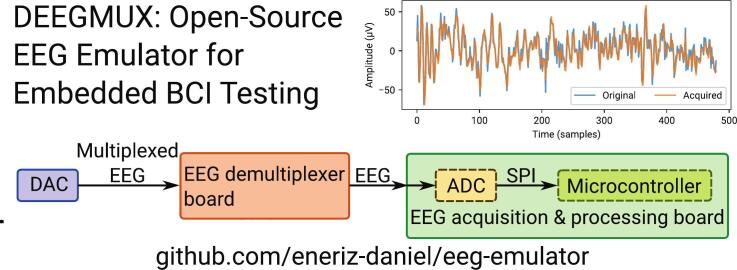

Specifications table

**Table:** 

Hardware name	DEEGMUX
Subject area	• Neuroscience
Hardware type	• Biological sample handling and preparation
Closest commercial analog	No commercial analog is available
Open source license	CERN-OHL-S-2.0 for hardware and GNU GPL v3.0 for software
Cost of hardware	145.80 €
Source file repository	https://doi.org/10.5281/zenodo.17488266

## 1 Hardware in context

Brain-Computer Interfaces (BCIs) represent a frontier in human–computer interaction, offering a direct communication pathway between the brain and external devices [1]. Electroencephalography (EEG) [2] stands out as a primary non-invasive modality for BCI systems, particularly for motor-imagery (MI) tasks, due to its high temporal resolution, portability, and relatively low-cost. The proliferation of powerful yet resource-constrained edge computing platforms has opened new possibilities for real-time BCI applications, from assistive technologies [3] to neurorehabilitation [4]. However, the successful deployment of these systems hinges on the effective implementation and validation of sophisticated machine learning models on-device.

Convolutional Neural Networks (CNNs) have emerged as a dominant architecture for processing raw EEG signals and classifying MI tasks due to their ability to automatically extract relevant spatio-temporal features [5], [6]. While the training of these data-hungry models is typically performed on powerful cloud or desktop systems using large, publicly available datasets, the ultimate performance and utility of the BCI system are determined by its real-world, on-device behavior. This presents a critical challenge: a model that performs well in simulation may fail when confronted with the noise, artifacts, and non-stationarity of real-world EEG data, compounded by the computational constraints of an embedded system.

Despite the growing need for hardware-in-the-loop (HIL) validation, there is currently no device, commercial or open-source, capable of replaying arbitrary, dataset-sourced EEG signals as physical analog outputs for embedded system testing. The problem can be approached from two directions, neither of which fully addresses this gap. On the hardware side, commercial EEG simulators such as the Netech MiniSim EEG 2000 [7] generate a fixed library of standard waveforms (alpha, beta, delta, etc.) for the calibration of clinical EEG recorders, but they cannot replay real subject-specific recordings from research datasets. On the software side, simulation toolboxes such as SEREEGA [8] and EEGSourceSim [9] generate synthetic EEG data numerically with realistic spectral and spatial properties, but they produce no analog output and therefore cannot exercise the real ADC front-end, quantization effects, and timing constraints of an embedded acquisition system. Furthermore, the specialized nature of EEG signal generation, which requires high-resolution, low-noise digital-to-analog converters (DACs) capable of operating in the microvolt range, together with the limited availability and high cost of scalable multichannel DACs suitable for biopotential applications, makes the development of custom EEG emulators particularly challenging.

To address this challenge, we have designed and built a low-cost, open-source EEG emulator. The hardware, named the DEEGMUX, is a scalable system designed to generate EEG signals corresponding to distinct MI tasks from a given dataset. The core of the device is an EEG demultiplexer able to demultiplex the signal from a DAC that outputs pre-programmed EEG signals multiplexed in time. By providing a stable, reproducible source of electrical signals that accurately represent real-world EEG, our hardware enables rigorous, automated, and HIL validation of on-device MI classification models. This tool facilitates a streamlined development pipeline for BCI researchers and engineers, allowing them to rapidly prototype and verify that their models operate as expected with real electronic inputs, paving the way for more robust and reliable BCI applications on the edge.

The terms “EEG demultiplexer” and “EEG emulation” were first introduced in [10], which defined these concepts: an EEG demultiplexer is a mentally emulated demultiplexer, that is, a device which uses mental actions to demultiplex a single EEG channel into multiple digital commands. In the paper, that definition was emulated in EEG, simulated in software and the EEG demultiplexer was used in a Brain-Computer Interface to control a physical robot arm, using a physical EEG signal. This line of work was further developed in [11]. In the present work, these terms are used in a different but complementary technical sense: rather than a conceptual framework, our EEG demultiplexer is a physical analog–digital mixed hardware circuit that distributes a time-multiplexed signal across parallel output channels, and EEG emulation refers to the electrical reproduction of database-sourced waveforms through a DAC for hardware-in-the-loop testing of embedded classification models, being applicable to BCIs, among other scenarios.

**Fig. 1:**
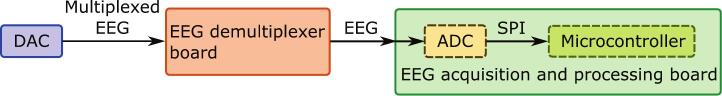
General scheme of the proposed system.

To complement the emulator, we also developed a dedicated data acquisition and processing PCB. This second board is designed to acquire the EEG signals generated by the emulator and serve as the edge device for our validation tests. It features the Texas Instruments ADS1299 [12], a well-regarded 8-channel, 24-bit analog-to-digital converter (ADC) widely validated in popular biopotential designs, including the OpenBCI headsets [13]. For on-board processing, we have integrated the Arduino Nano 33 BLE [14], a powerful and compact microcontroller unit capable of running lightweight CNNs and other machine learning models for real-time EEG signal processing and MI task detection [15]. The combination of these two custom-designed PCBs provides a complete, end-to-end hardware platform for the development, validation, and deployment of edge-based BCI systems, which is illustrated in Fig. 1. As we demonstrate in Section 7, this platform not only preserves signal fidelity at the analog level but also maintains the classification performance of a quantized EEGNet model, confirming its validity for end-to-end hardware-in-the-loop BCI testing.

## 2 Hardware description

### 2.1 EEG demultiplexer PCB

**Fig. 2:**
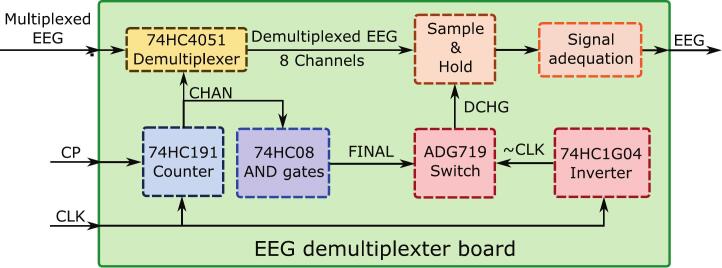
Scheme of the EEG demultiplexer board. The sample-and-hold and signal adequation blocks are placed for each of the channels.

**Fig. 3:**
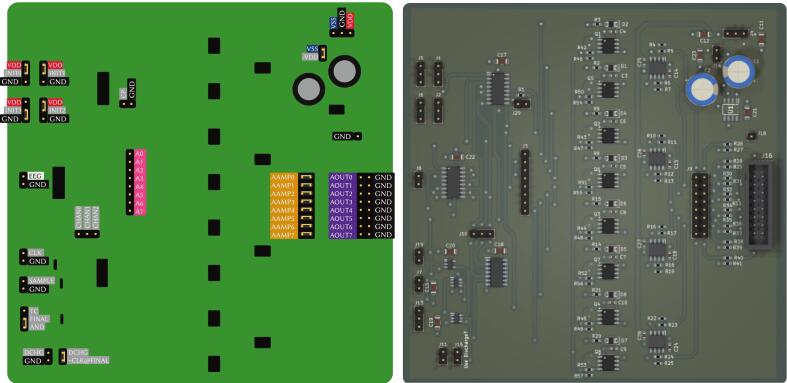
Pinout of the EEG demultiplexer board (left) and its 3D render (right).

The core of our EEG emulation system is a scalable demultiplexer PCB designed that converts a high-speed multiplexed input signal into a set of stable parallel EEG-scale outputs. Its block diagram is shown in Fig. 2, and the pinout and 3D render in Fig. 3. This board is built around the 74HC4051 8-channel analog multiplexer/demultiplexer from Nexperia [16].

An 8-channel base unit was selected as it aligns with the native channel count of both the 74HC4051 and the ADS1299 ADC used in the acquisition stage (Section 2.2), providing a hardware-consistent modular block. Moreover, eight channels (e.g., C_3_, C_z_, C_4_ and surrounding positions) comfortably cover the sensorimotor montages used in MI BCI, where competitive classification has been demonstrated with as few as three channels [17], and recent lightweight CNN classifiers targeting edge platforms have been successfully evaluated on 8-channel configurations [18], [19].

The 74HC4051 receives a single multiplexed analog input from the designated EEG pin and sequentially distributes it to one of eight individual output channels based on a 3-bit channel selection signal, the CHAN signal.

The sequential iteration through the demultiplexer's channels is managed by a 74HC191 4-bit binary counter from Nexperia [20], clocked by an external CLK signal. Only the three least significant bits of the counter are used for channel selection; the fourth bit is available to control additional cascaded demultiplexers (see scalability discussion below).. The counter features a parallel load input (CP) that allows the user to set a deterministic starting channel, ensuring predictable synchronization at startup.

**Fig. 4:**
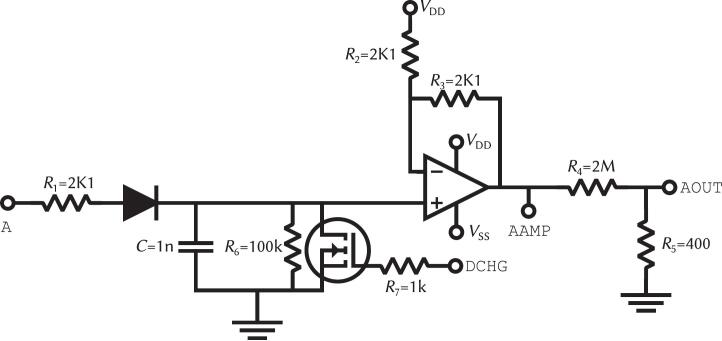
Sample-and-hold circuit and subsequent adequation for the demultiplexer outputs. The N-Channel MOSFET is a IRF8714 from Infineon, the diode is an 1N4148WS-G and the amplifier is a TL082 from Texas Instruments.

**Fig. 5:**
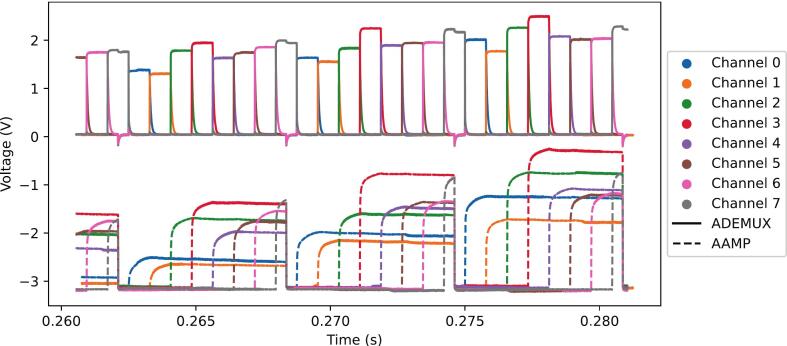
Demultiplexed signals before (ADEMUX) and after (AAMP) the sample-and-hold circuit.

To address the challenge of synchronized, multi-channel data acquisition, each demultiplexed channel incorporates a sample-and-hold circuit, shown in Fig. 4. This circuit holds each channel analog voltage long enough for the subsequent ADC to sample all channels synchronously. The circuit uses a 1N4148WS-G diode [21], selected for its low reverse recovery time which ensures clean isolation of the hold capacitor when the demultiplexer advances to the next channel, and a 1 nF capacitor, chosen to balance fast settling during the channel-active window against acceptable voltage droop during the hold interval.. The capacitor is buffered and its unipolar signal is converted to a bipolar, double-amplitude output using a TL082 operational amplifier [22] in a positive gain configuration with a DC offset. The TL082′s JFET input stage provides the high input impedance (1012 Ω) and low bias current (50 pA typical, 200 pA maximum) necessary to read the held voltage without appreciably discharging the capacitor, while its wide gain-bandwidth product (4 MHz) and high slew rate (13 V/µs) comfortably support the signal conditioning at the multiplexing clock rate. A comparison demonstrating how this circuit holds the signal until all channels are ready to be sampled is illustrated in Fig. 5.

Finally, a resistive voltage divider attenuates the output to the ±1 mV EEG range before being routed to the AOUT pins. An N-Channel MOSFET (IRF8714 from Infineon), controlled by the DCHG signal, periodically discharges the hold capacitors to prepare for the next acquisition frame.

**Fig. 6:**
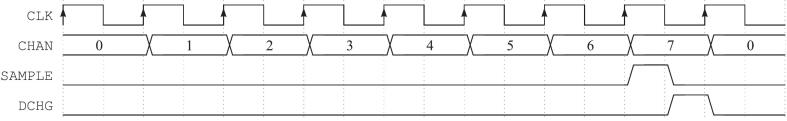
Timing diagram of digital signals of the demultiplexer board. CHAN bus is the selected channel of the demultiplexer. SAMPLE signal is raised when all channels are sampled. DCHG signal is used to discharge the capacitors to allow them to resample the next time frame.

A digital synchronization scheme coordinates the discharge and resampling cycle. The FINAL signal detects when the demultiplexer has completed a full pass through all eight channels. This triggers two actions: the SAMPLE signal, which indicates that all channels hold valid data and are ready for ADC acquisition, and the DCHG signal, which discharges the hold capacitors to prepare for the next frame. The default timing behavior of these control signals is illustrated in Fig. 6. The DCHG and SAMPLE signals are accessible via configurable jumpers, allowing users to adapt the timing logic to alternative acquisition schemes.

EEG data typically operates within a narrow physiological range, approximately ±1 mV. To ensure compatibility with standard commercial DACs, our board is designed to accept the multiplexed EEG input signal scaled to a unipolar range of 0–5 V. Following demultiplexing, the TL082 operational amplifiers not only isolate the signals but also convert them back to a bipolar range of ±5 V. A subsequent passive voltage divider network precisely attenuates this signal by a factor of 5000, effectively restoring it to the original ±1 mV EEG range. This process ensures the output signal is an accurate, scaled representation of the original biopotential data, ready for acquisition by an external ADC.

The board operates from a single-ended power supply, with VDD = 5 V and GND = 0 V. To provide the necessary bipolar voltage for the operational amplifiers, we have integrated a Texas Instruments LM2663 switched capacitor voltage converter [23]. This component is specifically designed to invert a positive input voltage, enabling the generation of a −VDD = −5 V rail. This negative supply, designated as VSS, is crucial for powering the TL082 operational amplifiers and allowing them to accurately process the bipolar EEG signals without clipping. Although switched capacitor converters inherently introduce switching ripple on their output rail, this noise is inconsequential in the present design. The TL082 amplifiers operate on signals in the 0–5 V range; any supply ripple coupled into the output is subsequently attenuated by the 5000:1 voltage divider that restores the signal to the ±1 mV EEG range. Should supply noise become a concern in more demanding configurations, the LM2663 could be replaced in a future board revision by a low-noise charge pump with integrated LDO output regulators, such as the Texas Instruments LM27762 [24], which maintains the same single-supply design philosophy while providing regulated, low-noise output rails (22 µV_RMS_).

The system can be straightforwardly expanded to any multiple of eight channels by simply cascading multiple 74HC4051 demultiplexers. This is achieved by extending the counter circuit to provide additional channel selection bits and by using the individual input enable pins of each multiplexer. This pin allows for the selective activation of a specific 74HC4051 chip, which can be controlled by a higher-order decoder circuit or directly by additional counter bits. This architecture ensures that only one demultiplexer's output is active at any given time, thereby maintaining a clean, single-channel signal path. This scalable design allows for the creation of multi-channel EEG emulators capable of simulating electrode arrays of varying sizes, from standard 8-channel setups to high-density systems, without requiring a complete redesign of the core circuitry. It should be noted, however, that scaling to higher channel counts (e.g., 16 or 32 channels) would proportionally increase the multiplexing clock rate, which may tighten the settling time budget of the sample-and-hold circuits and require careful timing evaluation at each stage; this has not been experimentally verified in the current work.

### 2.2 EEG acquisition and processing PCB

**Fig. 7:**
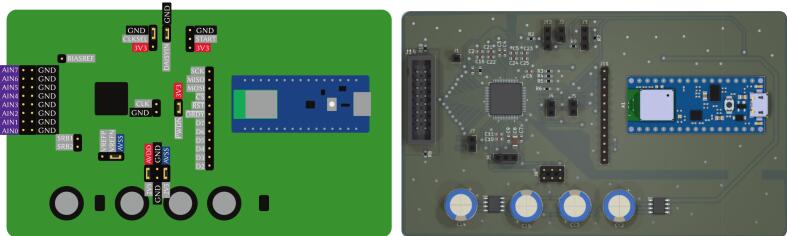
Pinout (left) and 3D render (right) of the EEG acquisition and processing board, comprising an ADS1299 ADC and an Arduino Nano 33 BLE.

Fig. 7 shows the pinout and the 3D render of the EEG acquisition and processing board. It features an 8-channel, 24-bit ADS1299 ADC connected to an Arduino Nano 33 BLE via a Serial Peripheral Interface (SPI) bus. For debugging purposes, the SPI pins (SCK, MISO, MOSI, and CS) are exposed. Additionally, the RESET and DRDY pins of the ADS1299 are accessible; the DRDY pin indicates when a new acquisition is ready to be read. Six digital pins of the Arduino (D2 to D7) are also exposed as general-purpose digital I/O for the PCB.

The ADS1299 is powered by a bipolar supply. We use two LM2663 switched capacitor voltage converters [23] to generate +2.5 V (AVDD) and −2.5 V (AVSS) from the Arduino's 5 V source, and connect them to the ADS1299 following the bipolar supply configuration described in the ADS1299 datasheet [12]., with decoupling capacitors as specified. The LM2663 was chosen for its minimal external component count and its ability to derive the required bipolar rails from a single USB-powered source without an external bench supply. Although switched capacitor converters inherently introduce output ripple, the ADS1299′s internal delta-sigma modulator and digital decimation filter provide strong power supply rejection which effectively suppresses supply-coupled interference within the EEG signal band. For applications requiring stricter supply noise specifications, a future revision of the board could incorporate a low-noise alternative such as the LM27762 [24], which combines a charge pump with integrated LDO regulators.

The START pin, exposed as an analog input on the ADS1299, allows for a hardware-triggered start of the acquisition. The CLK and CLKSEL pins permit the configuration of a custom clock for the ADS1299. If CLKSEL is connected to GND, the default 2.048 MHz internal clock is used. The VREFP and VREFN pins enable the use of a custom reference for the ADC. When VREFN is connected to AVSS, the ADS1299 can be digitally configured to use its internal 4.5 V reference.

The SRB1 and SRB2 pins are common reference points for the input channels and can be digitally programmed within the ADS1299. The BIASREF pin can be used for the bias signal feedback mechanism, which is commonly employed in professional EEG acquisition systems to reduce common-mode noise.

Furthermore, the DAISYIN pin on the ADS1299 is exposed, enabling the daisy-chaining of multiple ADS1299 chips. This feature allows for the acquisition of EEG signals from multiples of 8 channels, supporting scalable, high-density electrode arrays. An additional PWDN pin enables the user to power down the ADS1299 via a hardware signal, which is useful for power management in low-power applications.

This design allows the board to acquire both real and emulated EEG signals without compromising any of the ADS1299 key features. The choice of the ADS1299 was motivated by this versatility: as the industry-standard ADC for EEG acquisition (widely adopted in platforms such as OpenBCI [13]) it ensures that the board is not restricted to emulated signal testing but can equally serve as a front-end for real EEG recording, maximizing its utility for the BCI research community in general and our lab’s necessities in particular.

### 2.3 Complete system overview

**Fig. 8:**
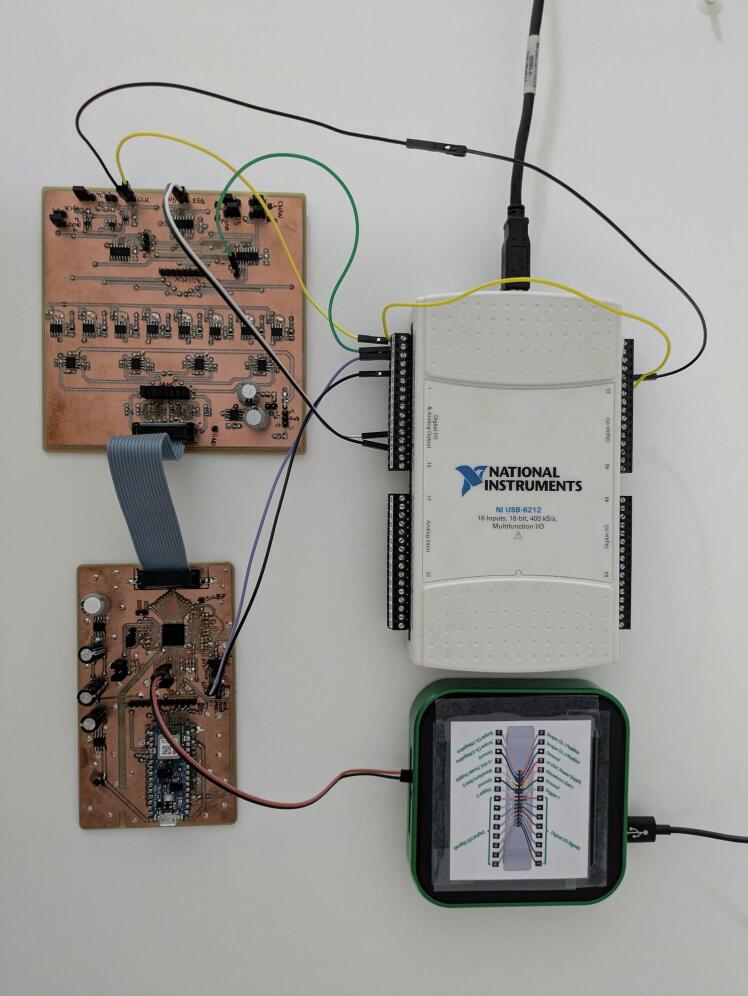
The image displays the complete EEG emulation and acquisition system. At the top left, the DAQ generates a multiplexed EEG signal, which is received by the EEG demultiplexer board at the bottom left. The resulting demultiplexed EEG signal is connected via a flat cable to the positive inputs of the ADS1299 on the EEG acquisition PCB, which is located at the bottom right. The Analog Discovery 3, at the top right, provides the custom clock signal for the ADS1299.

Fig. 8 provides an overview of the complete HIL validation system and its interconnections. The system's signal generation core is the National Instruments USB-6212 Data Acquisition (DAQ) board [25]. It features a 2-channel, 16-bit analog output capable of generating signals at a rate of 250 kSa/s in the ±10 V range. The timing for the multiplexed signal is precisely controlled by a clock signal generated using the DAQ's internal counter, CTR0, with its output available at pin PFI12. This clock signal is routed to pin PFI0, which serves as the timing reference for the analog output waveform on channel AO0.

The EEG signal from the USB-6212 DAQ is fed into our custom-designed EEG demultiplexer PCB. This board's demultiplexed output channels are then connected to the EEG acquisition and processing PCB, which handles the data conversion and on-device processing. To provide a custom clock frequency for the ADS1299 on the acquisition board, an external arbitrary function generator is used, specifically from an Analog Discovery 3 device.

## 3 Design files summary

All design files for the DEEGMUX system are openly available in the public repository located at https://github.com/eneriz-daniel/eeg-emulator. The electronic design was created using KiCad v9. The firmware for the Arduino Nano 33 BLE was developed in C++ using the PlatformIO ecosystem. Complementary host-side control software was developed in Python, consisting of two main files: one dedicated to managing the DAQ for signal generation, and the other serving as a Python wrapper to interface with the ADS1299 driver on the Arduino via serial communication.

The repository is logically structured into two primary components: eeg_emulation and eeg_acquisition, encompassing all hardware and software components. Table 1 provides a summary of the main files, their type, licensing, and specific locations within the repository.

**Table 1:** Main design files summary.

**Design file name**	**File type**	**Open source license**	**Location of the file**
eeg_demux.kicad_sch	Electronic schematic	CERN-OHL-S-2.0	https://github.com/eneriz-daniel/eeg-emulator/blob/master/eeg_emulation/design_files/eeg_demux.kicad_sch
eeg_demux.kicad_pcb	PCB layout	CERN-OHL-S-2.0	https://github.com/eneriz-daniel/eeg-emulator/blob/master/eeg_emulation/design_files/eeg_demux.kicad_pcb
daq.py	Python DAQ interface	GNU GPL v3.0	https://github.com/eneriz-daniel/eeg-emulator/blob/master/eeg_emulation/daq.py
eeg_acq.kicad_sch	Electronic schematic	CERN-OHL-S-2.0	https://github.com/eneriz-daniel/eeg-emulator/blob/master/eeg_acquisition/design_files/eeg_acq.kicad_sch
eeg_acq.kicad_pcb	PCB layout	CERN-OHL-S-2.0	https://github.com/eneriz-daniel/eeg-emulator/blob/master/eeg_acquisition/design_files/eeg_acq.kicad_pcb
ADS1299.cpp	Firmware source code	GNU GPL v3.0	https://github.com/eneriz-daniel/eeg-emulator/blob/master/eeg_acquisition/micro/src/ADS1299.cpp
ADS1299.h	Firmware header	GNU GPL v3.0	https://github.com/eneriz-daniel/eeg-emulator/blob/master/eeg_acquisition/micro/src/ADS1299.h
ADS1299Manager.cpp	Firmware source code	GNU GPL v3.0	https://github.com/eneriz-daniel/eeg-emulator/blob/master/eeg_acquisition/micro/src/ADS1299Manager.cpp
ADS1299Manager.h	Firmware header	GNU GPL v3.0	https://github.com/eneriz-daniel/eeg-emulator/blob/master/eeg_acquisition/micro/src/ADS1299Manager.h
ADS1299driver.py	Python driver	GNU GPL v3.0	https://github.com/eneriz-daniel/eeg-emulator/blob/master/eeg_acquisition/ADS1299driver.py

The project is organized to clearly separate the signal generation and acquisition components:

• eeg_emulation: Contains the files for the EEG signal emulation system, including the demultiplexer PCB and DAQ interface.
  o eeg_demux.kicad_sch: Schematic design for the 8-channel EEG signal demultiplexer.
  o eeg_demux.kicad_pcb: PCB layout for the EEG demultiplexer board, featuring careful analog signal routing and ground plane design.
  o daq.py: Python interface for controlling the NI USB-6212 DAQ device, handling signal generation and timing for the EEG emulation system. Also has the code to verify the EEG demultiplexer board.
• eeg_acquisition: Contains all files related to the EEG signal acquisition system, including the PCB design and ADS1299 driver.
  o eeg_acq.kicad_sch: Schematic design for the EEG acquisition board that interfaces an Arduino Nano 33 BLE Sense with the ADS1299 ADC, including power supply and digital connections.
  o eeg_acq.kicad_pcb: PCB layout design for the EEG acquisition board, optimized for low noise and proper digital-analog separation.
  o ADS1299.cpp and ADS1299.h: Low-level driver implementation for the ADS1299 ADC, handling SPI communication and basic register operations.
  o ADS1299Manager.cpp and ADS1299Manager.h: High-level interface for the ADS1299, providing functions for configuration, data acquisition, and serial communication.
  o ADS1299driver.py: Python wrapper for the ADS1299 firmware through serial port, enabling easy control and data acquisition from Python applications.

## 4 Bill of materials summary

The total cost of the open-source hardware system is summarized in the Bill of Materials Table 2, Table 3, which show the costs of each component in the EEG demultiplexer and EEG acquisition and processing boards, respectively. The EEG demultiplexer board has a total cost of 34.04 €, while the EEG acquisition and processing board has a total cost of 111.76 €. These costs are calculated excluding PCB manufacturing expenses.

**Table 2:** Bill of materials of EEG demultiplexer PCB.

**Designator**	**Component**	**Number**	**Cost per unit −currency**	**Total cost −** **currency**	**Source of materials**	**Material type**
IC1	74HC191D	1	0.47 €	0.47 €	Mouser – 74HC191D,653	Semiconductor
IC2	74HC4051D	1	0.26 €	0.26 €	Mouser – 74HC4051D,653	Semiconductor
IC3	74HC1G04GV	1	0.09 €	0.09 €	Mouser – 74HC1G04GV,125	Semiconductor
IC4	74HC08D	1	0.16 €	0.16 €	Mouser – 74HC08D,653	Semiconductor
IC5, IC6	ADG719BRTZ-REEL	2	3.46 €	6.92 €	Mouser – ADG719BRTZ-REEL	Semiconductor
IC9, IC12, IC15, IC18	TL082IDRQ1	4	0.83 €	3.32 €	Mouser – TL082IDRQ1	Semiconductor
U1	LM2663M	1	2.47 €	2.47 €	Mouser − LM2663M/NOPB	Semi-conductor
J1, J2, J5, J6, J13	Pin Header (1 × 3)	5	0.06 €	0.31 €	Diotronic – 40PY	Metal
J3	Pin Header (1 × 8)	1	0.17 €	0.17 €	Diotronic – 40PY	Metal
J4	Pin Header (1 × 3, Power Lines)	1	0.06 €	0.06 €	Diotronic – 40PY	Metal
J7, J8, J11, J14, J15, J19, J20	Pin Header (1 × 2)	7	0.04 €	0.29 €	Diotronic – 40PY	Metal
J9	Pin Header (2 × 8)	1	0.24 €	0.24 €	Diotronic – 2X40PY	Metal
J10	Pin Header (1 × 3, Channel Selection)	1	0.06 €	0.06 €	Diotronic – 40PY	Metal
J16	Omron XG4C-1631 Connector	1	0.94 €	0.94 €	Mouser – XG4C-1631	Composite
J18	Pin Header (1 × 1)	1	0.02 €	0.02 €	Diotronic – 40PY	Metal
Q1–Q8	IRF8714 MOSFET	8	0.76 €	6.08 €	Mouser – IRF8714TRPBFXTMA1	Semiconductor
D1–D8	Diodes 1N4148WS	8	0.09 €	0.72 €	Mouser – 1N4148WS-HE3-18	Semiconductor
C1, C2	Capacitors (47 µF, THT)	2	1.05 €	2.10 €	Mouser – ESU476M200AL3AA	Polymer
C3–C10	Capacitors (1 nF, 0603 SMD)	8	0.19 €	1.52 €	Mouser – AC0603FRNPO9BN102	Ceramic
C11–C23	Capacitors (100 nF, 1206 SMD)	10	0.10 €	1.05 €	Mouser – AC1206KKX7R0BB104	Ceramic
C14–C28	Capacitors (100 nF, 0603 SMD)	8	0.12 €	0.96 €	Mouser – AC0603KPX7R9BB104	Ceramic
R1	Resistor (10 kΩ, 0603 SMD)	1	0.02 €	0.02 €	Mouser – AC0603FR-7W10KL	Ceramic
R2–R25	Resistors (2.1 kΩ, 0603 SMD)	24	0.03 €	0.77 €	Mouser – AA0603FR-072K1L	Ceramic
R26, R28, R30, R32, R34, R36, R38, R40	Resistors (2 MΩ, 0603 SMD)	8	0.01 €	0.05 €	Mouser – AC0603FR-072ML	Ceramic
R27, R29, R31, R33, R35, R37, R39, R41	Resistors (400 Ω, 0603 SMD)	8	0.02 €	0.15 €	Mouser – AF0603FR-07402RL	Ceramic
R42–R45, R50–R53	Resistors (100 kΩ, 0603 SMD)	8	0.02 €	0.18 €	Mouser – AC0603FR-7W100KL	Ceramic
R46–R49, R54–R57	Resistors (1 kΩ, 0603 SMD)	8	0.02 €	0.15 €	Mouser – AF0603FR-131KL	Ceramic
Jumpers	−	14	0.04 €	0.56 €	Diotronic – AKSPL	Metal
Cable sockets	−	2	1.97 €	3.94 €	Diotronic – 609.1641	Metal
Flat cable	−	0.01 m	1.39 €/m	0.01 €	Diotronic – CP16	Metal

**Table 3:** Bill of materials of EEG acquisition and processing PCB.

**Designator**	**Component**	**Number**	**Cost per unit – currency**	**Total cost – currency**	**Source of materials**	**Material type**
A1	Arduino Nano 33 BLE	1	19.87 €	19.87 €	Mouser − ABX00071	Non-specific
A1 (Socket)	Pin Socket (1x15)	2	0.37 €	0.74 €	Diotronic – 40SYA	Metal
U1	ADS1299	1	76.95 €	76.95 €	Mouser – ADS1299IPAG	Semiconductor
U2, U3	LM2663M	2	2.47 €	4.94 €	Mouser – LM2663M/NOPB	Semiconductor
J1	Pin Header (1 × 1)	1	0.02 €	0.02 €	Diotronic – 40PY	Metal
J2, J4, J5, J7	Pin Header (1 × 2)	4	0.04 €	0.17 €	Diotronic – 40PY	Metal
J3, J6, J12	Pin Header (1 × 3)	3	0.06 €	0.19 €	Diotronic – 40PY	Metal
J8	Pin Header (2 × 3)	1	0.09 €	0.09 €	Diotronic – 2X40PY	Metal
J11	Pin Header (1 × 12)	1	0.25 €	0.25 €	Diotronic – 40PY	Metal
J14	Omron XG4C-1631 Connector	1	0.94 €	0.94 €	Mouser – XG4C-1631	Composite
C1	Capacitor (1.5 nF, 0603 SMD)	1	0.09 €	0.09 €	Mouser – AC0603KRX7R9BB152	Ceramic
C2, C3, C5-C7, C9, C10, C21, C23	Capacitors (1 µF, 0603 SMD)	9	0.13 €	1.61 €	Mouser – AC0603KRX7R6BB105	Ceramic
C4, C16, C22, C24, C25	Capacitors (100 nF, 0603 SMD)	5	0.12 €	0.60 €	Mouser – AC0603KPX7R9BB104	Ceramic
C8	Capacitor (100 µF, 1206 SMD)	1	0.47 €	0.47 €	Mouser – CC1206MKX5R5BB107	Ceramic
C12-C15	Capacitors (47 µF, THT)	4	1.05 €	4.20 €	Mouser – ESU476M200AL3AA	Polymer
R1	Resistor (1 MΩ, 0603 SMD)	1	0.23 €	0.23 €	Mouser – AR0603FR-071ML	Ceramic
R2-R7, R9	Resistor (10 kΩ, 0603 SMD)	7	0.02 €	0.16 €	Mouser – AC0603FR-7W10KL	Ceramic
Jumpers	−	6	0.04 €	0.24 €	Diotronic – AKSPL	Metal

It should be noted that the complete hardware-in-the-loop system additionally requires a general-purpose device capable of providing a hardware-timed analog output and a synchronous clock signal (in our setup, an NI USB-6212), as well as a function generator for providing an external clock to the ADS1299 when a non-standard data rate is required (in our setup, an Analog Discovery 3). The NI USB-6212 is overspecified for this application, as the system only requires a single analog output channel and one counter/timer output. The DEEGMUX is designed to interface with any device that can provide these two signals; in principle, a low-cost microcontroller such as the Raspberry Pi Pico (∼4 €), combined with an external SPI DAC (e.g., Microchip MCP4921 at ∼3 €) and using its programmable I/O peripherals for clock generation, could serve as a significantly more affordable signal source, though this configuration has not been experimentally validated. For reproducible results, the NI USB-6212 remains the validated and recommended signal source for this system.

## 5 Build instructions

### 5.1 PCB Fabrication and preparation

The custom PCBs for the EEG emulator system—the EEG Demultiplexer Board and the EEG Acquisition and Processing Board—can be fabricated using the standard Gerber files generated from their respective layout files (eeg_demux.kicad_pcb and eeg_acq.kicad_pcb) within KiCad v9. These files are accepted by all major PCB manufacturers. For our prototypes, we utilized the internal manufacturing service provided by the Servicio de Instrumentación Electrónica of the Servicio General de Apoyo a la Investigación-SAI, Universidad de Zaragoza.

Upon receiving the manufactured PCBs, a crucial preliminary step is quality assurance. Perform a thorough visual inspection to check for any manufacturing defects, such as solder bridges, lifted pads, or misaligned vias. Following the visual check, use a multimeter to test the continuity of all essential traces, pads, and vias. The provided KiCad layout files serve as the definitive reference for verifying the intended connections between all points on the board.

### 5.2 Assembly process and recommended tools

Once the PCB integrity is verified, the assembly process can commence. Our prototypes were fully hand-soldered, but any reliable soldering technique, such as reflow or selective soldering, can be used, provided it ensures robust electrical connections between all components and their respective pads. Any necessary files for standard assembling process can be generated from the Kicad project files.

The designs utilize a mix of Surface-Mount Technology (SMD) and Through-Hole Technology (THT) components. The generally recommended assembly approach is sequential to minimize the chance of damaging already-placed components or obstructing access to smaller pads:

• SMD Components First: Begin by soldering the smallest SMD components, then progress to the larger ones (e.g., resistors/capacitors before ICs).
• THT Components Last: Complete the assembly by soldering all THT components (e.g., headers, connectors, through-hole ICs).

For hand-soldering SMD components, we recommend the following technique: apply a small amount of flux to the pads, use a fine-tip soldering iron (0.5–0.8 mm tip), and use fine-gauge solder wire (0.3–0.5 mm). This technique facilitates quick, clean joints and reduces the risk of creating solder bridges.

List below contains the materials and tools we used for our assembly process.

• Soldering station with closed-loop thermal control
• Solder wire
• Flux
• Desolder braid and solder sucker
• Tweezers (fine, anti-static)
• PCB holder
• Magnifier or microscope
• ESD protection: wrist strap, mat
• Multimeter for continuity checks

After completing the soldering, place the pin jumpers as shown in Fig. 3, Fig. 4, Fig. 5, Fig. 6, Fig. 7 for standard operation mode.

### 5.3 Safety and security instructions

Given that the acquired signal involves low-voltage electronics intended for eventual bio-potential research, adhering to strict safety and component security procedures is mandatory during assembly.

I. Electrical Safety
• Ventilation: Always work in a well-ventilated area or use a fume extractor when soldering to avoid inhaling flux fumes, which are harmful irritants.
• Temperature Control: Use a soldering station with closed-loop thermal control to maintain a consistent tip temperature, preventing accidental burns and minimizing component thermal stress.
• First Power-Up: Before connecting the assembled board to the power source, perform a final, meticulous check for solder bridges using a multimeter. Initial power-up should be done with a current-limited power supply if possible, to prevent immediate damage in case of a short circuit.
II. Electrostatic Discharge (ESD) Protection

The integrated circuits, particularly the high-precision ADS1299 ADC and the fine-pitch microcontroller, are sensitive to ESD damage.

• Grounding: Always wear an ESD wrist strap connected to a grounded ESD mat. The mat should cover the entire working surface.
• Component Handling: Handle components, especially ICs, only by their bodies and only when necessary. Store sensitive components in their original anti-static packaging until they are ready to be soldered.

### 5.4 Verification

#### 5.4.1 EEG demultiplexer board

To verify the proper assembly and functionality of the EEG Demultiplexer Board, we have provided a validation routine using the host PC and the NI DAQ. The Python script located at daq.py is designed to generate the multiplexed EEG signal via one analog output channel of the DAQ while simultaneously acquiring the demultiplexed signals using eight analog input channels.

Since the primary outputs of the EEG demultiplexer are scaled down to the sensitive ±1 mV EEG range, which may be below the optimal acquisition range of the DAQ's analog inputs, we recommend connecting the DAQ's analog input channels to the AAMP pins (see Fig. 3). The AAMP pins are located before the final voltage divider network, providing an output in the ±5 V range. This approach maximizes the signal-to-noise ratio during verification.

If the verification script fails to execute or the acquired signals show significant distortion compared to the original, generated waveform, the PCB requires detailed troubleshooting. We recommend following the structured signal flow and control logic checks outlined below, referencing the components specified in the eeg_demux.kicad_sch schematic. A digital oscilloscope and a logic analyzer will be necessary for effective debugging.

1. Multiplexed Signal Path: Check that the high-speed multiplexed input signal is successfully reaching the analog input pin of the 74HC4051 demultiplexer (IC2) via the appropriate connector (J8).
2. Clock and Counter Functionality: Verify the operation of the channel counter logic.
  a. Check the CLK signal (J19) from the DAQ to ensure the 74HC191D counter (IC1) is clocked.
  b. Monitor the channel selection output CHAN (J10) to confirm the counter is progressing sequentially.
3. Counter Reset Configuration: Verify the counter's initialization.
  a. Check that the counter starts counting from the seventh channel (CHAN = 7) when the parallel load signal CP (J20) is asserted (low).
  b. Ensure that jumpers J1, J2, J5, and J6 are correctly bridged to set the data input to '7′ for parallel loading.
4. Demultiplexer Operation: Validate the core demultiplexing process by cross-referencing three key signals: the input multiplexed signal (J8), the CLK signal (J19), the CHAN value (J10), and the individual demultiplexed analog signals (A signals at J3).
5. Synchronization and Discharge Logic:
  a. Check the logic path for the end-of-cycle marker: Verify that pin 2 of the connector J13 is correctly connected to pin 6 of IC4, and that this point is high when the counter reaches CHAN = 7.
  b. Check the discharge activation: Ensure the pins in J15 are correctly shorted to enable the capacitor discharge mechanism.
  c. Verify the timing of the discharge signal: Check that the DCHG signal (J11) is high during the second half of the clock period when the counter is selecting CHAN = 7.
6. Sample-and-Hold Output: Examine the analog outputs at the AAMP signals (J9).
  a. Confirm that these signals properly settle and maintain a stable value during their respective acquisition clock period.
  b. Verify that the signal is actively shorted to GND (discharged) when the DCHG signal is high, preparing the capacitor for the next acquisition frame.

#### 5.4.2 EEG acquisition and processing board

Verifying the EEG Acquisition and Processing Board requires validating both the microcontroller firmware and the sensitive analog front-end (ADS1299). A fundamental test is running the live plotting feature provided in the Python host-side driver, ADS1299driver.py.

First, upload the appropriate firmware, located in the micro/ folder, onto the Arduino Nano 33 BLE microcontroller. Part of the board is powered by the + 5 V reference from the Arduino Nano 33 BLE USB connection. Crucially, ensure the VUSB pads on the reverse side of the Arduino board are soldered shut (bridged), as the VUSB feature is disabled by default. Identify the serial port identifier for your Arduino on the host PC and configure this port in the main section of the ADS1299driver.py script before execution.

If the ADS1299driver.py driver fails to initialize, communicate, or acquire data correctly, follow the structured troubleshooting guide below. Use the eeg_acq.kicad_sch as your primary reference, and consult the ADS1299 datasheet [7] for detailed information on the ADC's operation and SPI programming.

1. Reference Voltage Check: Ensure the ADS1299 has proper connections to use its internal reference. This is typically achieved by shorting pins 1 and 2 at J6.
2. Clock Source Check: Connect the CLKSEL pin (J12) to GND to enable the use of the ADS1299′s default 2.048 MHz internal clock.
3. Power Supply Validation: Verify that the ADS1299 is properly powered. Use a multimeter to check the voltages at both the digital and analog power sources on the board, confirming the correct +2.5 V (AVDD) and −2.5 V (AVSS) rails are present and stable.
4. SPI Communication Test: The core of the issue often lies in communication failure. Connect a logic analyzer to the exposed SPI pins (MISO, MOSI, SCK, and CS at J11) to monitor traffic. Verify that the Arduino is sending correct commands and receiving valid responses from the ADS1299.
5. Data Ready Signal: Check the DRDY signal (pin 6 at J11). This active-low pin should transition low after every successful conversion, indicating that new data is available to be read by the microcontroller. If this signal remains high or transitions incorrectly, the ADS1299 is not completing its acquisition cycle.

## 6 Operation instructions

### 6.1 EEG demultiplexer board operation

Operating the EEG Demultiplexer Board requires proper power supply and signal synchronization:

1. Power Connection: Connect a reliable power source to the board's power input pins (J4 in eeg_demux.kicad_sch). The +5 V rail is mandatory. The necessary negative reference, −5 V can be generated on-board by the LM2663M voltage converter by simply shorting the pins at J14, which activates its output.
2. Signal Input: Connect the host-generated multiplexed EEG signal and its high-frequency clock to the board's EEG and CLK pins (Fig. 3), respectively.
3. Counter Initialization: Before the first signal transmission, the user must ensure the on-board counter is properly reset by momentarily driving the CP pin low. This action prepares the demultiplexer for the start of the multiplexed sequence.

The value loaded on reset is determined by the logic levels at the INIT0 to INIT3 pins. The default jumper settings (J1, J2, J5, J6) load the value corresponding to the eighth channel, which causes the counter to begin its sequence from channel 0 on the first clock cycle after CP is asserted low.

By default, the DCHG signal is connected to the ∼After these initial steps, the board will begin operation as described in Section 2.1, demultiplexing the input signal until the multiplexed EEG stream and its corresponding clock signal cease.

CLK@FINAL pin, representing the inverted clock when the FINAL pin is high. The FINAL pin is linked to the output of an AND gate (IC4) that detects when the counter reaches channel 7. Consequently, DCHG is high during the second half of the clock cycle when the eighth channel is active, discharging the hold capacitors. Conversely, SAMPLE goes high during the first half of this same clock cycle, serving as the synchronization marker for external acquisition systems. These defaults can be reconfigured via jumpers J13, and J15 as needed for specific applications.

To ease the signal generation process from a host PC, we've included the EEGMux Python class within the daq.py file. This class encapsulates all the necessary logic to control the NI USB-6212 DAQ, automatically configuring it to:

• Generate the multiplexed EEG signal via analog output AO0.
• Generate the high-speed clock signal via counter CTR0.
• Utilize the general-purpose pin PFI1 to send the required reset signal to the board's counter.

### 6.2 EEG acquisition and processing board operation

The EEG acquisition and processing board is powered using the 3.3 V and 5 V sources from the Arduino Nano 33 BLE, from which AVDD and AVSS are derived using the LM2663M voltage converters, to power up the board, you only need to plug in a powered micro-USB in the Arduino Nano 33 BLE.

We have included in our repo the micro/ folder, which contains all the firmware to be uploaded in the Arduino Nano 33 BLE. It contains all the code to communicate with both, the ADS1299 though SPI and a host PC through serial port. The serial port offers a low-level interface with the board, since the firmware has been coded to allow programming the ADS1299 from the host PC to control its behavior at run time and also get the acquisition from the ADS1299 in real time. On top of this, a Python driver is provided in ADS1299driver.py to allow the programmatic control of the ADS1299 from a host PC. Table 4 includes a summary of all the functions that are made available as serial commands and as methods in the driver.

**Table 4:** Summary of the ADS1299 programming functions included in the driver.

Function	Command syntax	Python driver method	Arguments
Enable channel	e[channel]	enable_channel(channel)	channel: Integer between 1 and 8
Disable channel	d[channel]	disable_channel(channel)	channel: Integer between 1 and 8
Set PGA gain for channel	g[channel][gain]	set_gain(channel, gain)	channel: Integer between 1 and 8gain: Integer in (0, 1, 2, 3, 4, 6, 8, 12, 24). 0 means the PGA is bypassed
Set input mode for channel	i[channel][mode]	set_input_mode(channel, mode)	channel: Integer between 1 and 8mode: String in (‘normal’, ‘shorted’, ‘test’)
Enable data continuous mode	z	rdatac()	−
Stop data continuous mode	x	sdatac()	−
Start streaming data	a	start_streaming()	−
Stop straming data	s	stop_streaming()	−
Single read	k	single_read()	−
Set batch read	n[n_samples]	set_batch_read(n_samples)	n_samples: Number of samples to acquire in batch
Get batch read	m	get_batch_read()	−
Set data rate	f[data_rate]	set_data_rate(data_rate)	data_rate: Integer in (16000, 8000, 4000, 200, 1000, 500, 250). Values in Sa/s.
Set reference	r[ref][mode][channel]	set_reference(ref, mode, channel)	ref: String in (‘SRB1′, ‘SRB2′)mode: Bool (enable/disable) channel: Integer between 1 and 8. Is only used for SRB2.
Set single-shot mode	k[enable]	single_shot_mode(enable)	enable: Bool (enable/disable)
Set test signal	t[amplitude][frequency]	test_singal(amplitude, frequency)	amplitude: String in (‘1′, ‘2′)frequency: String in (‘slow’, ‘fast’, ‘dc’)
Print ADS1299 registers	?	print_all_registers()	−

The EEG Acquisition and Processing Board is designed for simplified power and control.

**Fig. 9:**
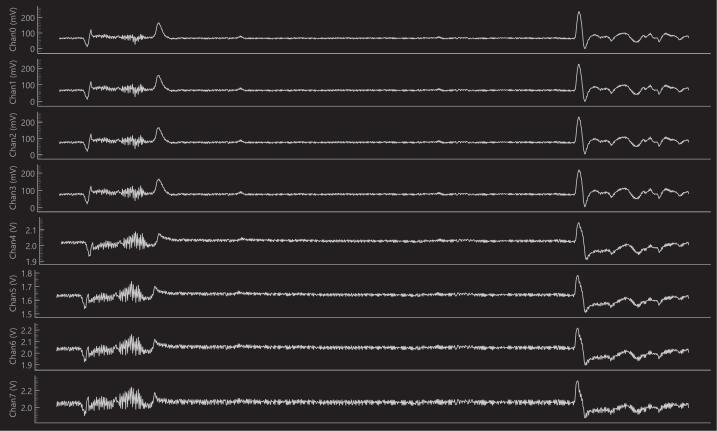
Plot window generated by live_plot.

1. Power-Up: The board is powered entirely by the Arduino Nano 33 BLE. Power is supplied simply by plugging a powered micro-USB cable into the Arduino. The 3.3 V and 5 V sources from the Arduino are used as the primary rails, from which the on-board LM2663M converters derive the required AVDD and AVSS rails to power the analog front-end.
2. Firmware Loading: The micro/ folder in the repository contains all the C++ firmware that must be uploaded to the Arduino Nano 33 BLE. This code handles all communication: both the low-level SPI interface with the ADS1299 and the high-level serial communication with the host PC.
3. Host PC Control: The firmware provides a low-level serial interface, allowing users to program the ADS1299′s behavior (e.g., sample rate, gain) at run time and stream acquisition data in real time. Building upon this interface, the ADS1299driver.py Python driver provides a robust, programmatic control layer from the host PC. Table 4 summarizes the available functions that are mapped between serial commands and driver methods.
4. Live Plotting: The Python driver includes a live_plot method, which enables real-time visualization of the signals being acquired by the ADS1299 on the host PC screen (Fig. 9). To ensure fast and smooth processing of this data stream, the PyQtGraph Python package [26] is used.

### 6.3 Complete system operation

The full HIL validation is orchestrated by the test.py Python script, which centralizes the control logic for both the signal generation (DAQ) and the acquisition (ADS1299) boards.

This script programs the ADS1299 to use the Read Data Continuous mode, which sets the ADC to continuously sample its inputs while the START pin (available at J3 in eeg_acq.kicad_sch) is held high. Also sets the lowest data rate, 250 Sa/s, and configures the PGAs with highest gain, 24, across all channels. This ensures best quality acquisition of the EEG samples.

A critical aspect of the system is the precise synchronization required due to the settling time inherent to the ADS1299’s Read Data Continuous mode. This settling time means the first valid sample is not acquired immediately after the START pin goes high. To ensure the first valid acquisition occurs precisely when the demultiplexer board has successfully presented all eight channels of the EEG signal, a fine-grained synchronization mechanism is necessary.

**Fig. 10:**
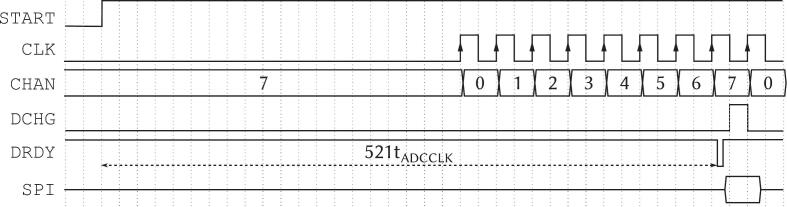
Inter-board synchronization with the START signal. t_ADCCLK_ is the period of the ADS1299′s clock.

The ADS1299 datasheet [12] specifies the exact number of ADC clock periods required for the settling time at any given data rate. For the selected 250 Sa/s data rate, the settling time is 521 ADC clock periods (t_ADCCLK_). The test.py script leverages a general-purpose pin on the DAQ, PFI2, to precisely control the START signal timing. By setting the START signal high at an exact time instant before the target acquisition window, we compensate for the known 521 clock period delay. This ensures that the ADS1299′s first stable conversion occurs at the moment all eight demultiplexed EEG channels are stable, achieving the required hardware synchronization across the entire system. The timing of these signals is shown in Fig. 10.

## 7 Validation and characterization

### 7.1 Signal generation and synchronization

To validate the hardware designs and the software implementations we used the Physionet EEG Motor Movement/Imagery dataset [27], [28]. This dataset contains 64-channel EEG recordings from 109 subjects, sampled at 160 Hz. Since our prototype is 8-channel system, we selected a subset with electrodes AF_3_, AF_4_, C_3_, C_Z_, C_4_, P_3_, P_4_, PO_Z_, locations according to the 10–10 international system.

To ensure synchronous acquisition at the desired 160 Sa/s rate with the ADS1299 operating in Read Data Continuous mode, we employed an external clock signal. The ADS1299′s lowest data rate configuration with its internal 2.048 MHz clock, f, yields a minimum data rate, DR, of 250 Sa/s. To achieve the desired data rate, DR’, of 160 Sa/s, the required external clock frequency, f’, was calculated using the following relationship:

(1) $$f\prime =\frac{\mathrm{DR}\prime }{\mathrm{DR}}f$$

Substituting the values, the required external clock frequency, f’, is 1.311 MHz. This external clock signal was generated using the arbitrary waveform generator of an Analog Discovery 3, as illustrated in Fig. 8.

### 7.2 Characterization results

**Fig. 11:**
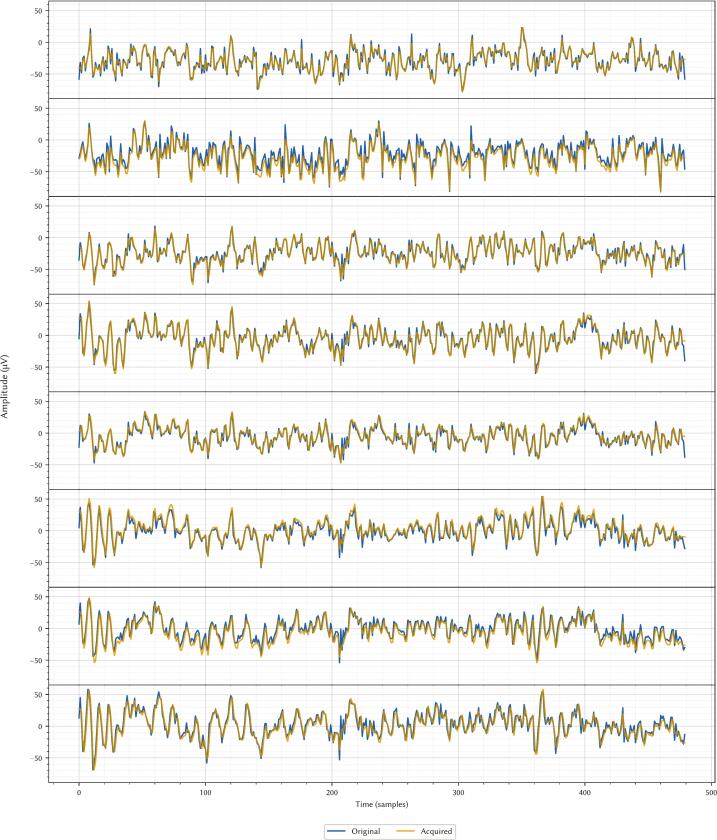
Example of EEG acquisition. Original data is in blue, while acquired data is in gold. (For interpretation of the references to colour in this figure legend, the reader is referred to the web version of this article.)

We successfully emulated and acquired the entire 8-channel subset of the Physionet dataset. Fig. 11 shows a qualitative comparison between the original digital data and the signal acquired by our system. The close visual correlation demonstrates the high fidelity of the signal generation and acquisition chain.

The quantitative fidelity of the system was assessed using three common metrics: Mean Squared Error (MSE), Mean Absolute Error (MAE), and Signal-to-Noise Ratio (SNR). These metrics were computed for each channel in each sample of the dataset and then averaged. The metrics used are defined as follows, where x and y are vectors of length N representing an original and acquired sample for a given channel, and x_i_ and y_i_ their i-th elements:

(2) $$MSE\left(x,y\right)=\frac{1}{N}\sum_{i=0}^{N-1}{\left(x_{i}-y_{i}\right)}^{2}$$

(3) $$MSE\left(x,y\right)=\frac{1}{N}\sum_{i=0}^{N-1}{\left(x_{i}-y_{i}\right)}^{2}$$

(4) $$SNR\left(x,y\right)=10\;{log}_{10}\left(\frac{\frac{1}{N}\sum_{i=0}^{N-1}{x_{i}}^{2}}{\frac{1}{N}\sum_{i=0}^{N-1}{\left(x_{i}-y_{i}\right)}^{2}}\right)$$

The averaged results across all channels and samples are MSE of (1.7 ± 4.7)·10^-10^ V^2^, a MAE of (7.5 ± 7.7)·10^-6^ V and an SNR of 16 ± 6 dB. The low MSE and MAE values confirm that the acquired signal closely matches the original digital data. The SNR of 16 dB confirms that the signal's power is significantly higher than the introduced noise (quantization and electronic), validating the high-fidelity design of the demultiplexer and the acquisition system for bio-potential signals. These system-level metrics inherently capture all noise sources present in the signal chain, including quantization noise, electronic noise, and any power supply ripple from the LM2663 switched capacitor converters on both boards, confirming that the overall noise budget remains adequate for biopotential signal acquisition. Notably, neither the ADS1299 nor the NI USB-6212 DAC are the dominant contributors to this noise floor: the ADS1299′s input-referred noise at the operating configuration (gain 24, 250 Sa/s) is 0.14 µV_RMS_ [12], and the DAC's quantization noise referred to the EEG output scale is approximately 0.035 µV_RMS_, both well below the observed error. The system-level SNR is therefore primarily determined by the analog demultiplexing and signal conditioning stages, including charge injection and switching transients in the sample-and-hold circuits, voltage droop during the hold interval, and component tolerances in the 5000:1 resistive attenuation network.

### 7.3 BCI model validation

To demonstrate the system's practical utility for its intended application, we evaluated the impact of the HIL signal chain on CNN-based MI classification. Specifically, the acquired data were used to evaluate an EEGNet model configured for 8 channels, quantized to 8-bit integers with TensorFlow Lite following the methodology in [18]. The same quantized model was evaluated on both the original digital data from the PhysioNet dataset and the signals acquired through the DEEGMUX system.

The evaluation yielded an accuracy difference of (−0.3 ± 5)% between the hardware-acquired and original digital data, where the negative sign indicates a marginally higher mean accuracy on the acquired signals. This negligible difference demonstrates that the 16 dB system SNR does not meaningfully degrade CNN classification performance and confirms the system's suitability for HIL BCI testing. The result validates that models trained and benchmarked on digital datasets can be reliably tested through the DEEGMUX's analog signal chain without introducing performance-altering artifacts.

Taken together, the characterization and classification results presented in this section confirm that the DEEGMUX provides a faithful and practical hardware-in-the-loop testing platform. By offering an open-source, reproducible system that bridges the gap between numerical simulation and real-world deployment, the DEEGMUX enables BCI researchers to rigorously validate embedded classification models under realistic electronic conditions before committing to costly clinical EEG recordings. This lowers the barrier to iterative model development on edge devices and accelerates the transition from laboratory prototypes to deployable BCI systems, being applicable to any other scenario involving low-amplitude biosignals.

## CRediT authorship contribution statement

**Daniel Enériz:** Writing – original draft, Software, Methodology, Investigation, Formal analysis. **Diego Antolín:** Writing – review & editing, Validation, Methodology. **Nicolás Medrano:** Writing – review & editing, Project administration, Funding acquisition, Conceptualization. **Belén Calvo:** Writing – review & editing, Supervision, Project administration, Funding acquisition.

## Declaration of competing interest

The authors declare the following financial interests/personal relationships which may be considered as potential competing interests: Belen Calvo reports financial support was provided by Agencial Estatal de Investigación y FEDER UE PID2022-138785OB-I00. Daniel Eneriz reports financial support was provided by Gobierno de Aragón under Grant BOA20201210014. If there are other authors, they declare that they have no known competing financial interests or personal relationships that could have appeared to influence the work reported in this paper.
